# A comparison of three methods to generate a conceptual understanding of a disease based on the patients’ perspective

**DOI:** 10.1186/s41687-017-0013-6

**Published:** 2017-12-19

**Authors:** Louise Humphrey, Thomas Willgoss, Andrew Trigg, Stephanie Meysner, Mary Kane, Sally Dickinson, Helen Kitchen

**Affiliations:** 1 Clinical Outcomes Solutions, LLC, Unit 68, Basepoint, Shearway Business Park, Shearway Road, Folkestone, Manchester, CT194RH UK; 2 DRG Abacus, Manchester, UK; 3 Formerly of DRG Abacus, Manchester, UK; 4 University of Liverpool, Liverpool, UK; 5 Concept Systems, Inc, Ithaca, NY USA; 6 National Ankylosing Spondylitis Society, London, UK; 7 Adelphi Values, Cheshire, UK

## Abstract

**Background:**

The Food and Drug Administration patient-reported outcome (PRO) guidance provides standards for PRO development, but these standards bring scientific and logistical challenges which can result in a lengthy and expensive instrument development process. Thus, more pragmatic methods are needed alongside traditional approaches.

**Methods:**

Partnering with the National Ankylosing Spondylitis (AS) Society, we compared three methods for eliciting patient experiences: 1) concept elicitation (CE) interviews with 12 individuals with AS, 2) “group concept mapping” (GCM) with 16 individuals with AS, 3) a social media review (SMR) of AS online chatrooms. Three conceptual models were developed and compared to explore data breadth/depth, as well as the practicalities and patient-centeredness.

**Results:**

Overlap in concepts was observed between conceptual models; 35% of symptoms were identified by all methods. The SMR approach identified the most concepts (*n* = 23), followed by CE interviews (*n* = 18), and GCM (*n* = 15). Eight symptoms were uniquely identified using GCM and SMR. Eliciting in-depth data was challenging for SMR as detail was not always provided. Insight into the relationships between symptoms was obtained as a “concept map” in GCM, via effective probing within interviews, and through the subject’s descriptions in SMR. Practical investment varied; CE interviews were the most resource intensive, whereas SMR was the least. Individuals in GCM and CE interviews reported high engagement.

**Conclusions:**

Primary CE interviews achieved the greatest depth in conceptual understanding of patient experience; however, novel methods (GCM, SMR) provide complementary approaches for identifying measurement concepts. Each method has strengths and weaknesses and should be selected based on specific research objectives.

**Electronic supplementary material:**

The online version of this article (10.1186/s41687-017-0013-6) contains supplementary material, which is available to authorized users.

## Background

Over the last 30 years the role of the patient in clinical drug development has shifted from fractional to central. The publication of the Food and Drug Administration’s (FDA) guidance and the European Medicines Agency’s (EMA) reflection paper on patient-reported outcomes (PRO) measurement within clinical drug development are both testament to the enhanced role of the patient in regulatory strategy and decision-making [1–3]. However, while regulatory guidance helps pharmaceutical companies and instrument developers understand the evidence requirements for generating new PRO measures, they also bring with them considerable scientific and logistical challenges [4]. For example, interview techniques that elicit rich, deep, and meaningful patient insights are recommended in the FDA’s PRO guidance, yet they bear heavy resource investment. While the improved standards for PRO development results in more robust, valid, and reliable measurement, the time and cost associated with developing new PRO tools using methods recommended in the guidance has limited the speed at which new tools become available for use [5, 6]. As a consequence, there is growing recognition in the field of PRO development that the logistical and practical barriers to instrument development need to be addressed through exploring novel methodologies that retain scientific rigor but promote greater and quicker access to effective new measurement tools [4]. While the potential of such methods have been discussed between regulatory, industry and academic representatives [4, 7], no consensus on their regulatory acceptability has been obtained, in part due to a lack of empirical comparisons between such methods in the context of PRO development.

### Study aims

There is limited research exploring the value of traditional (i.e. as set out in FDA guidance) PRO content development methods in comparison with other novel methods [7, 8]. Our research group sought to further the science by conducting an empirical assessment of the relative benefits and limitations of two emerging methods for eliciting and collecting the patient perspective relative to traditional approaches described in the PRO guidance.

## Methods

Three methodological approaches were selected for evaluation in this study. First, face-to-face concept elicitation (CE) interviews were identified as the ‘traditional’ approach [1]. Second, in light of the increasing use of online technology, the next approach selected was to collect data from social media sources [4]. Third, in light of the FDA’s interest in mixed methods (i.e. intentional mixing of qualitative and quantitative data) an approach called “Group Concept Mapping” (GCM) was identified that utilizes an online platform to elicit and quantify patient insights [9]. An overview of the three methods is presented in Table 1.

**Table 1:** Overview of methods under evaluation in this study

	CE Interviews	Social Media Review	GCM
Type of data collected	Qualitative	Qualitative	Qualitative and quantitative
Design of data collection method	Primary, prospective	Secondary, retrospective^a^	Primary, prospective
Depth of data collected	Deep, rich	Varies depending on platform	Shallow
Breadth of data collected	Specific to study aims	Broad	Narrow
Ability to probe and explore new areas of interest	High - interviewer interacts with subject, facilitates discussion and probes around key areas of interest	Medium - dependent on existing data. If discussion not present in SM thread, cannot probe further	Low – only pose one or two questions (or prompts) and responses are dependent on the quality of the prompt and the instructions to subjects
Availability of clinical/background data	High – although depends on recruitment approach	Low	High – although depends on recruitment approach
Ability to confirm diagnosis	High – although depends on recruitment approach	Medium (typically self-confirmed diagnosis only)	High – although depends on recruitment approach
Level of burden on subjects	High	Low	Low
Level of burden on researcher	High	Low	Medium
Time and cost burden	High	Low	Low
Scientific acceptance/ best practice	High	Low	Mixed (widely applied in other fields but less so for outcomes research)
Regulatory support	High	Mixed (depends on purpose of research)	Mixed (broadly supportive of mixed methods approaches that utilize online technologies)

*CE* concept elicitation, *GCM* group concept mapping

^a^A social media review may also be performed prospectively but for the current study a retrospective approach was employed

Each method was employed to elicit a conceptual understanding of the symptom experience from the patient’s perspective. Such work is often conducted in the early planning stages of a clinical outcomes assessment strategy for clinical trials, where a broad understanding of salient disease-related concepts is first sought before selecting those of most interest to target through subjective assessment. Procedures for each method are described below.

### Recruitment of subjects

For the CE interviews and GCM exercise, it was necessary to prospectively recruit subjects to participate in the research. We collaborated with the National Ankylosing Spondylitis Society (NASS), a charity based in the United Kingdom that supports individuals living with ankylosing spondylitis (AS) - a chronic, inflammatory disease causing painful joints and stiffness [10, 11]. CE interview and GCM participants were recruited through the NASS member database. All NASS members in Greater Manchester (for CE interviews) and Greater London (for GCM) who had a registered email address with the charity were invited to the study via email.

Individuals who responded to the invitation were required to read a subject information sheet and asked to read, sign, and return an informed consent form if they were willing to participate. We aimed to recruit at least 12 subjects to both study arms; this sample size was targeted with the aim of achieving ‘conceptual saturation’ [1, 12]. In qualitative research where there is a well-defined, focused research question involving a group of fairly homogenous individuals, this sample size has previously been sufficient to achieve conceptual saturation [12, 13].

Self-reported demographic and clinical data were collected: gender, age (years), and severity of AS on a 0 to 10 scale (where higher scores equate to greater disease severity). Diagnosis of AS was also self-reported by participants. Since subjects were recruited via the NASS database, centralized ethics approval (e.g. National Health Service) was not required for this study. However, guidance for Good Clinical Practice to protect human research subjects was followed, including the requirement for written informed consent and ensuring anonymity and confidentiality of data [14].

**Fig. 1:**
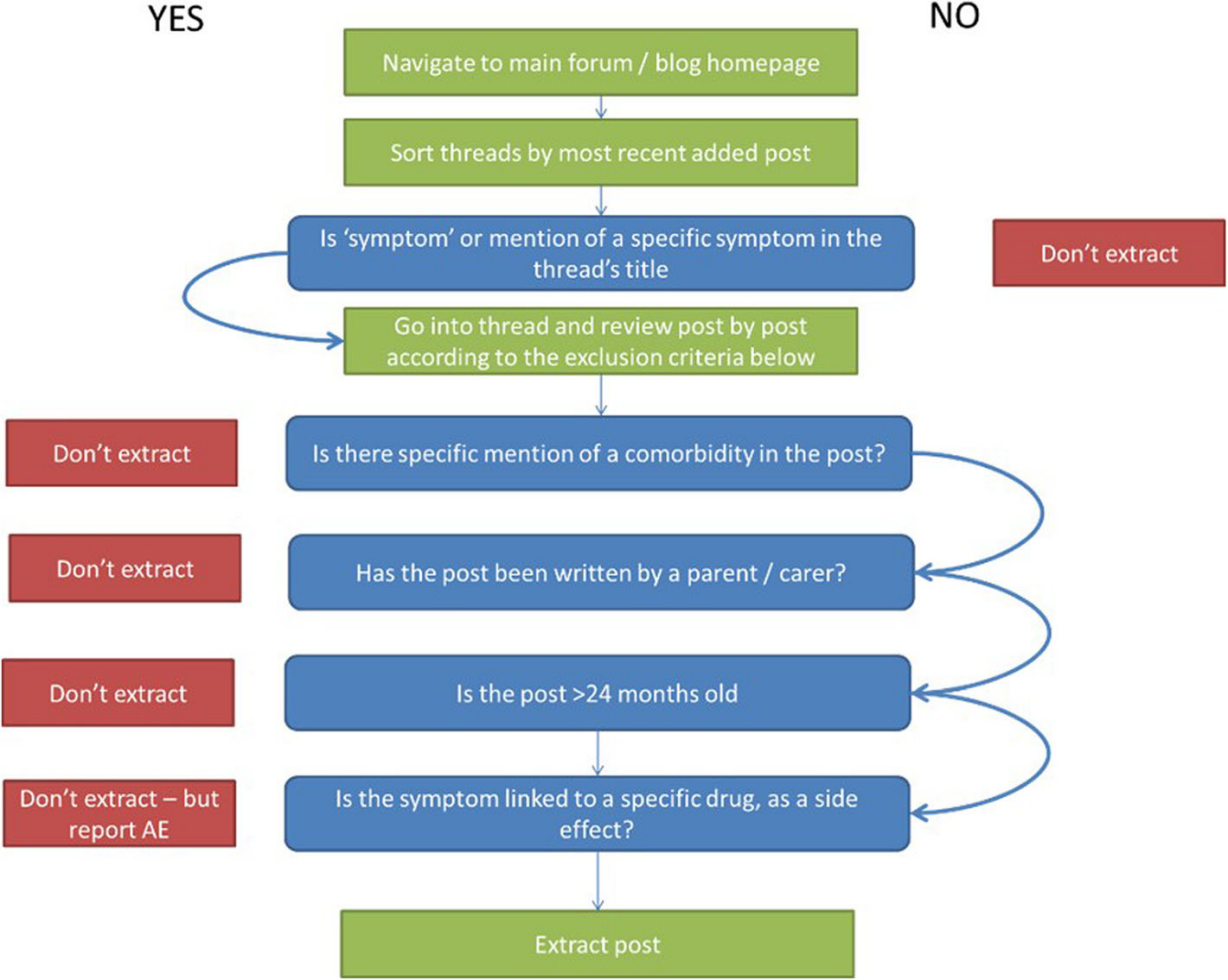
Social media review search strategy and selection process

We gathered social media entries from pre-existing social media sources. Given the secondary nature of these data, an a priori protocol was developed that pre-specified search terms, steps to identify relevant data and extraction rules [15]. An internet search was undertaken (using www.google.co.uk) using the terms (“ankylosing spondylitis OR AS”) AND (“forum OR chatroom”) to identify AS-related social media forums. Forums were specifically targeted (rather than single-authored blogs or social networking sites) due to the naturalistic discussions between patients on such sites. The first 20 results were reviewed and each website assessed for suitability based on the inclusion/exclusion criteria (Table 2). If more than five websites were identified as relevant, priority was given to those websites that 1) were endorsed or sponsored by an AS charity, support group, or trust and 2) provided demographic and/or clinical information alongside each post. Once forums were identified, a selection process for posts was employed (Fig. 1) to ensure that recent entries were captured, targeting discussions on disease-related symptoms.

**Table 2:** Inclusion and exclusion criteria used to assess suitability of websites initially identified in social media search

Inclusion	Exclusion
Meets definition of ‘forum’ (ie, where members post responses to a thread and interact with each other)	Member login required, to protect assumed privacy
An AS-specific section of the website exists	Any entry in the terms of service / terms and conditions / copyright information / privacy policy sections of the website prohibit the use of content hosted on the website
Members of the website are primarily patients, rather than care-givers	
English language	
Content has been added in the last 12 months	

*AS* ankylosing spondylitis

### Data collection

Data collection for each method was conducted independently, where one researcher followed each methodology.

#### Concept elicitation interviews

Subjects were invited to attend a 60-min CE interview to talk about their experiences of living with AS. A semi-structured interview guide was used by a trained, qualitative researcher to encourage discussion around the subject’s AS symptom experiences. During the open discussion, subjects were asked to use a ‘body map’ (diagram of a human body) to mark areas where they experienced AS symptoms. This allowed the subject to discuss their personal experiences using their own language and terminology. At the end of the interview, subjects were invited to provide written feedback of their experience as an interview participant.

#### Social media review

Similar to the identification of suitable posts, the identification of relevant information to be extracted was specified in an a-priori protocol. In addition to the post itself, extracted information included the thread title, date of post, and username, as well as gender, age, location, and the year of AS diagnosis, if available. Due to the nature of the social media data, a clinician-confirmed diagnosis of AS could not be obtained for each author of a post. The protocol specified that 100 eligible posts would be extracted for coding and analysis. The usernames associated with each post were monitored to ensure that no more than five posts were collected from each unique author.

### Group concept mapping

Group concept mapping [9] intentionally collects and integrates both qualitative and quantitative data, and as such, is a mixed-method approach. This study used online GCM software (GlobalMAX™). As a data collection method, GCM has been widely applied in organizational and policy settings, especially public health, and is an emerging method in patient reported outcomes research [9, 16–22]. For the GCM exercise, data were generated over three participant stages as described by Kane and Trochim (2007) [9]: 1) “Concept Generation”, 2) “Sorting” and, 3) “Rating” (a fourth stage, “Mapping,” is the analysis stage). Once recruited, each eligible subject was sent an email with a link to a webpage hosting the online GlobalMAX™ software. Subjects were presented with the following research prompt *“A symptom of my ankylosing spondylitis is…”* They were then asked to generate as many responses as they wished to this prompt (Concept Generation). The total list of responses from all subjects was consolidated and any duplicates were removed. This resulted in a list of patient-authored symptom descriptors that represented the experiences of the entire sample. Once the list of symptom descriptors had been finalized, each subject was sent a second email with a link to the study webpage. Subjects were asked to individually sort and label all symptom descriptors into groups using a drag-and-drop table top interface, based on how he or she considered them to be related to one another (Sorting). Once each participant had sorted all symptom descriptors into groups of his or her design, each subject rated the severity (from ‘not at all severe’ to ‘very severe’), frequency (from ‘never’ to ‘always’), and impact (from ‘not at all bothersome’ to ‘very bothersome’) of each symptom descriptor on a 0 to 4 response scale (Rating). Following data collection, each subject was invited to provide feedback on their experiences of taking part in the GCM exercise.

### Data analysis

Data elicited from each method were analyzed separately to explore the symptoms reported by study subjects. To minimize any potential biases, three researchers from the study team independently analyzed the findings derived from each separate methodology. A further two researchers resolved any conflicts during analysis. The concurrence between findings was assessed to examine the scientific value of each method in terms of the breadth and depth of insight generated. Researcher and participant reflections were then amalgamated to consider the practical and logistical advantages and limitations of each method.

### Procedures for data analysis

#### Concept elicitation interviews

All CE interviews were audio-recoded and transcribed *verbatim*. Transcripts were imported into a qualitative software package (ATLAS.ti Version 7) [23] and subject to thematic analysis [24]. To meet the study objectives, analysis was focused on identifying the key themes relating to AS symptom experiences. Inductive codes (i.e. codes generated from the content of the interview transcripts) were assigned to words and phrases relating to symptoms. The code list was refined throughout the data collection process and the final code list was reviewed and validated by the project lead. Codes were also organized into domains and sub-domains as common themes emerged in the data. Content and thematic analysis methods were used to present counts and verbatim examples of subject responses during the CE interview. Where possible, differences between concepts according to demographic and clinical background were explored.

#### Social media review

Qualitative data generated from the SMR were subject to thematic analysis, as per the CE interview data. Note that full quotes of posts are not reported within this paper to protect the identity of their authors.

#### Group concept mapping

The qualitative and quantitative data generated through GCM were analyzed using multivariate techniques within the GlobalMAX™ software, as described by Kane and Trochim (2007) [9]. First, data generated in the “Sorting” task were collated into a similarity matrix, which presents the frequency with which symptom descriptors were grouped together. Next, multidimensional scaling of the similarity matrix was performed to generate co-ordinates for each symptom descriptor, where the proximity of descriptors indicates their similarity to each other. These co-ordinates were subjected to hierarchical cluster analysis to aggregate proximal symptoms into clusters, i.e., conceptual domains. Finally, the average severity, frequency, and impact rating for each symptom descriptor and domain were calculated.

From the above analyses, a series of “Maps” were generated displaying subject-generated symptom descriptors, the broader domains, and ratings on the various dimensions.

### Determining the scientific value of the tested methods

A conceptual model of AS symptoms was created by independent researchers, each model included the conceptual domains and sub-domains that arose in each independent dataset, thus allowing comparisons to be made between the three methods in terms of its ability to capture all relevant and important symptoms of AS. The conceptual models derived using CE interview and SMR data were determined based on the researcher’s interpretation of the qualitative findings and compared to a conceptual model derived from the symptoms and domains within the maps generated through GCM task. The degree of overlap, as well as any discrepancy between the conceptual models provided critical insight in terms of the value and credibility of each methodology evaluated in this study.

### Determining the practical advantages and limitations of the tested methods

In the context of PRO research, choice of study design can be heavily influenced by cost, time, and labour limitations; hence the impetus to develop more flexible and pragmatic research methods. We conducted a comparison of the cost, time, and labour investment required for each of the three methods to understand the relative practical benefits and limitations of each approach.

### Understanding the subjects’ perspective of the tested methods

Feedback from subjects in the GCM and CE interviews was used to evaluate each method from the participant perspective. Analysis of subject feedback focused on three main aspects: 1) task complexity, 2) level of engagement, and 3) overall experience. Participant feedback could not be ascertained in the SMR due to its secondary nature.

## Results

### Sample results

Twelve participants completed a CE interview, 16 participants completed the GCM exercise, and 100 blog posts from two websites (www.spondylitis.org and www.kickas.org) were analyzed. Demographic and self-reported severity of AS symptoms are presented in Table 3.

**Table 3:** Demographic results

	CE interviews (*N* = 12)	Social Media Review^a^ (*N* = 100)	GCM Exercise (*N* = 16)
Age, mean (range)	53 (32–78)	28-58^b^	53 (33–70)
Gender, % male	80	41^c^	78
Severity (0–10 NRS in past 2 weeks), mean (range)	2.4 (0–5)	*Not known*	3.8 (0–8)

*CE* concept elicitation, *GCM* group concept mapping, *NRS* Numeric Rating Scale

^a^Demographic and clinical data was not always provided in posts

^b^Data available in 23/100 posts

^c^Data available in 34/100 posts

### Comparison of methods: scientific learnings

**Fig. 2:**
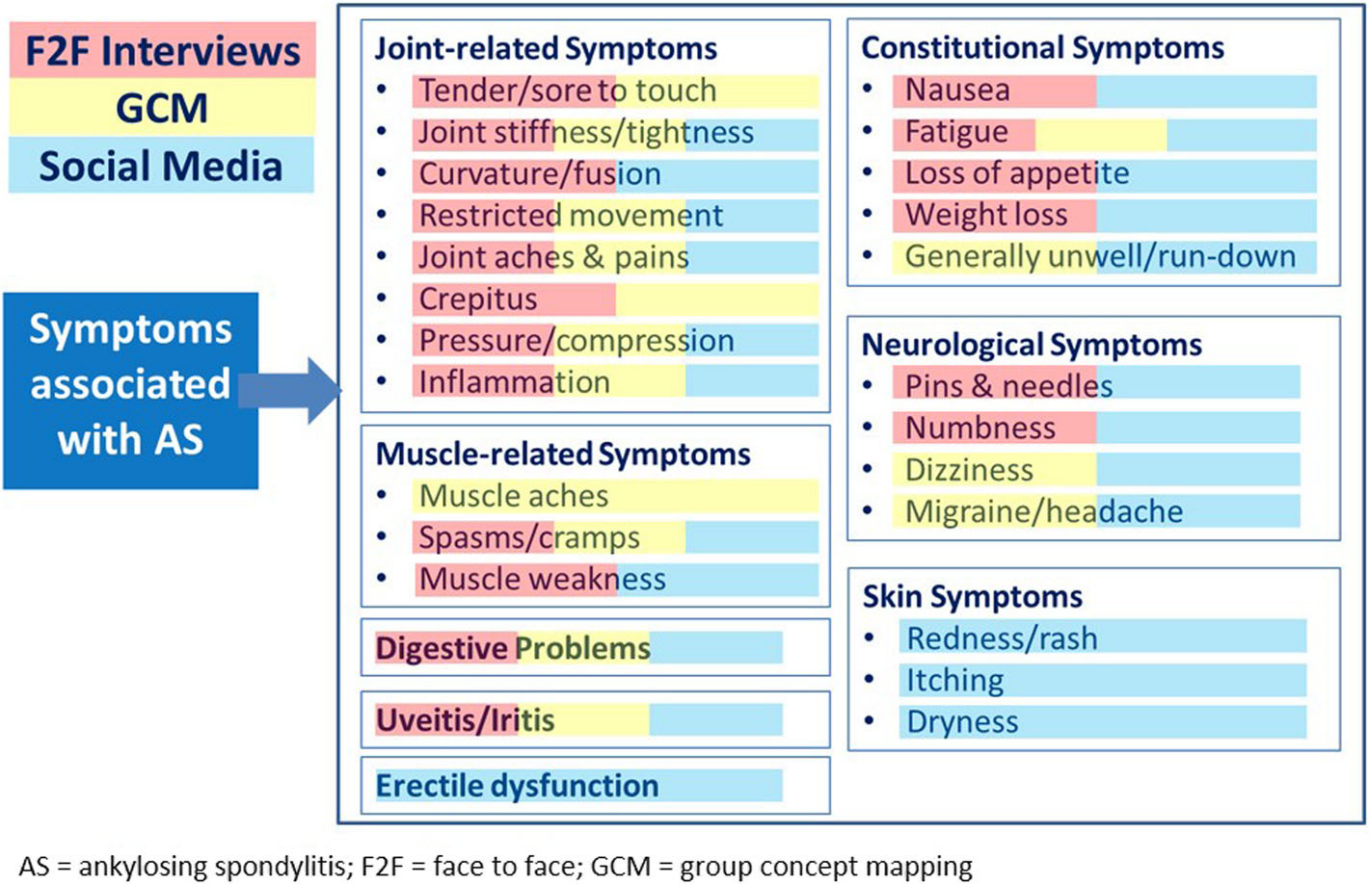
A combined conceptual model of AS symptoms identified based on all three methods

The first objective was to examine how effective each method was at identifying the symptoms of AS. Analysis of all data collected from the three approaches highlighted 26 symptom concepts as relevant and important to those living with AS. These 26 symptoms are conceptualized into eight symptom clusters or domains, as presented in a combined conceptual model (Fig. 2).

Despite the differences between the three methods tested (in terms of data type, depth, and design), there was overlap in the concepts identified by each method (Fig. 2). Just over one-third (35%) of symptom concepts (*n* = 9) were captured by all three approaches. The SMR identified the highest number of concepts (88% of all symptoms; *n* = 23), CE interviews and GCM captured 69% (*n* = 18) and 58% (*n* = 15) of all symptoms concepts, respectively.

Unique symptom concepts were identified in the GCM exercise and within the SMR. “Loss of appetite,” “erectile dysfunction” and all three skin symptoms (“redness/rash”, “itching”, “dryness”) were identified in the SMR only; whereas “muscle aches” was reported in the GCM exercise only. Interestingly, symptoms that may be classed as sensitive or “socially embarrassing” (such as erectile dysfunction and skin symptoms) were only identified in the SMR. It has been noted previously that the anonymous nature of an online chatroom or forum, for example, provides individuals with the opportunity to report experiences that they may not feel comfortable discussing in an interview or focus group setting [18].

The next objective was to examine how effective each method was at identifying the relative importance of the different symptoms identified. This was most challenging for SMR data (Table 4). Information on the severity or impact of each symptom was reliant on chance descriptions within posts and therefore the opinion of the whole sample may not be fully or accurately captured. In contrast, both GCM and the CE interview methods allowed for specific and direct exploration of the *severity*, *frequency*, and *bother* associated with each symptom for all subjects. Based on CE interview data, the most frequent, severe, and impactful symptoms were “fatigue”, “shoulder pain”, and “lower back pain”, respectively. Fatigue was the most prominent symptom across the methods employed, featuring in all three categories within GCM as well as being the most frequent symptom in the CE interviews.

**Table 4:** Comparison of methods according to the most severe, frequent, and bothersome AS symptoms

	Most Frequent	Most Severe	Most Impactful	How was this Identified?
CE interviews (*N* = 12)	Fatigue Neck pain Lower back pain	Shoulder pain Lower back pain Neck pain	Lower back pain Shoulder pain Neck pain	Specifically probed during interview as each symptom was mentioned
Social media review (*N* = 100 posts)	Unable to determine based on postsreviewed	Fatigue Lower back pain	Unable to determine based on posts reviewed	If described within chatroom post.
GCM (*N* = 16) (*Average rating data)*	Fatigue (2.4) Stiff neck (2.4) Restricted spine movement (2.2)	Fatigue (2.6) Stiff neck (2.4) Stiff back (2.1)	Fatigue (1.6) Neck pain (1.4) Stiff neck (1.4)	Based on ‘ratings’ data (stage 3 of GCM)

*AS* ankylosing spondylitis, *CE* concept elicitation, *GCM* group concept mapping

**Fig. 3:**
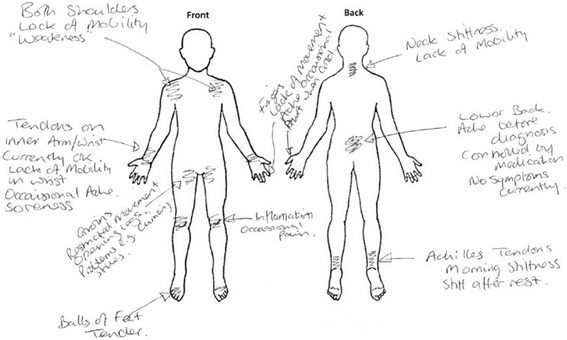
Body mapping exercise as completed by Participant-05

Another objective was to examine how effective each method was in generating an in-depth understanding of the subjects’ experience of their disease. To facilitate the generation of more detailed and descriptive data, we used a ‘body map’ technique in the CE interviews. An example of one participant’s body map this is shown in Fig. 3.

*“Right the front, I think my head is fine, definitely here, all round here…..”*

*“I: And if you can just talk me through as you’re shading in what you’re shading?”*

*“My neck and my collarbones are always quite swollen, so I would say all of this, all this area here actually, and my shoulders are always pretty bad round here. This bit is fine, arms are fine, joints on my fingers can be bad, so I’ll shade those in on both sides. A lot of this has gone, …... yes, to on the front of my hand, my joints. Then the hip area, that’s really bad, and probably down the spine, but it’s probably more at the back than the front. Knees are bad. I never really tell anybody this, because you don’t really want to moan about it”.(Participant-02, Female, 49 years old).*

*“The main symptom I had at the point I was diagnosed, it was an ache in my lower back and it’s a very unspecific thing, but I have to say I started taking anti-inflammatories and that pretty much went away, and now I wouldn’t say that I get any problems with my lower back really, I’d say that’s completely controlled by the medication.”* (Participant-05, Male, 50 years old).

Detailed descriptions of symptoms were also identified in the review of chatroom posts. However, these descriptions were typically less detailed than those elicited in the CE interviews and were without any opportunity for further probing.

Due to the nature of the GCM approach, detailed descriptions from subjects were not obtained. However, a more in-depth understanding of subjects’ experience of symptoms was obtained in the form of the maps generated using multivariate analyses conducted using the GlobalMAX™ software. The maps not only illustrated which symptoms were most important, but also showed how subjects collectively perceived the relationship between symptoms and more general symptom domains.

**Fig. 4:**
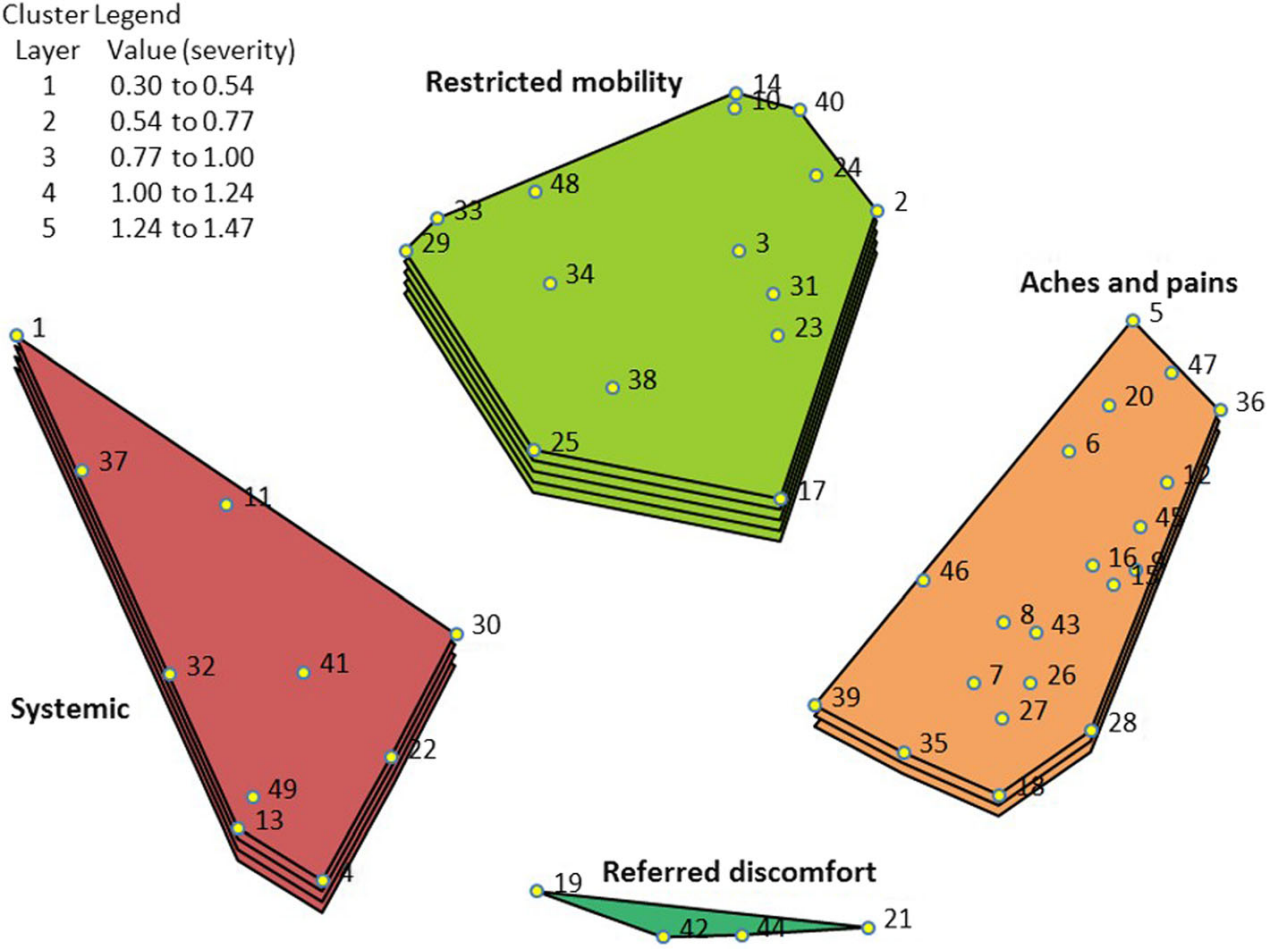
3D map detailing the 4-cluster solution, rated at the domain level by severity

Subjects participating in GCM considered the symptoms of stiff neck, stiff back, and restricted movement in the spine to be related closely; based on labels provided by participants this domain was best labelled “restricted mobility” (Fig. 4). Neck pain, muscle aches, and back pain were also grouped together and labelled “aches and pains.” Fatigue, generally feeling unwell/run-down, and problems with the digestive system were considered related within a single domain labelled “systemic” symptoms. Finally, symptoms of uveitis and pressure in the head were grouped together and labelled “referred discomfort”.

The map also showed the relatedness between symptom descriptors within a specific domain. The statements corresponding to each point on the map are provided in Additional file 1 Table S1. For example, within the “aches and pains” symptoms domain, the close positioning of “intermittent pain in ankles” (point 7) and “intermittent pain in Achilles/heel” (point 26) indicated that these were seen by participants as highly related symptoms. Conversely, although “fatigue” (point 1) and “clicking sounds in shoulder” (point 30) were both labelled “systemic” symptoms, they were perceived to be quite distinct symptoms within the domain (by the relative distance between points). Indeed, “clicking sounds in shoulders” was likely to have been grouped with “difficulty extending arms” (point 25) by some subjects, hence the proximity between points on the map, despite being grouped into different domains.

### Comparison of methods: practical learnings

Overall, the CE interviews required significantly more time than both the SMR and GCM (35 days versus 5 days and 12 days, respectively). The additional time in the CE interviews was largely driven by subject recruitment and scheduling of interviews (Table 5).

**Table 5:** Comparison of the time invested for each method evaluated

Task	CE interviews (*N* = 12)	Social media review (*N* = 100 posts)	GCM (*N* = 16)
Development of study protocol and other materials^a^	5 days	1 day	1 day
Recruitment of subjects	15 days (includes time to schedule interviews)	0 days	5 days
Data collection	10 days	1 day	5 days
Data analysis	5 days	3 days	1 day
TOTAL	35 days	5 days	12 days

*CE* concept elicitation, *GCM* group concept mapping

^a^If approval were sought for this study from the UK’s NHS ethics review board, this would have added approximately 6–12 weeks to the study timelines. Timeline for ethics approval varies depending on the country where the research is to be conducted

### Comparison of methods: subject perspective

In terms of task complexity, almost all subjects reported that neither methodology was difficult to complete. For the CE interviews, the in-depth interview guide and experienced interviewer ensured that subjects understood what they were being asked to do throughout the interview. Similarly, the GCM task was considered clear and straightforward: “*I liked the drag and dropping. It was really easy to use, and was clear,*” and “t*he grouping exercise was interesting.*” This is important since there was no ‘live’ researcher to assist the subject if there were problems or questions. That said, one subject reported that the GCM instructions were unclear: “*I wasn’t really sure what to name the piles at first,*” and felt that “*researcher support would have been helpful.*”

Participant engagement was reported to be high for both the CE interviews and GCM exercise, with some subjects participating in the GCM commenting that they “*appreciated the chance to lead the research.*” Overall, subjects reported positive experiences in taking part in both elements of the research, with interview subjects also noting that they found it a “*cathartic*” experience.

## Discussion

### Which method was the most scientifically credible?

In terms of breadth of insight, the SMR approach elicited the most symptom concepts, including five symptoms that were not identified in either of the other two methods. We hypothesize that the anonymity provided by an online setting may have allowed subjects to feel they could describe socially embarrassing symptoms more readily [7]. In contrast, participants may not have felt comfortable talking about these experiences in more ‘identifiable’ settings (i.e., a face-to-face interview). An alternative explanation is that the five ‘unique’ social media symptoms were not critical or common to AS and therefore not reported in the CE interviews or GCM exercise. Additional research is required to explore this further.

While all seven domains included in the combined conceptual model were captured to some extent by the SMR, the data was limited in terms of depth of understanding. Due to the secondary nature of the social media data, we were unable to follow-up or probe further into the subjects’ experiences. Notably, this is characteristic of secondary data regardless of its source; further investigation using primary data elicited through social media is warranted. In contrast, we explored subjects’ AS symptoms in greater depth in the CE interviews and the GCM exercise. Specifically, we focused on understanding which symptoms were most common, most bothersome, and most severe from the subjects’ perspective. Details regarding the frequency and severity of symptoms are an important source of input at a later stage when constructing items (i.e. wording, response options and recall). A major criticism of using secondary social media data in this type of research is related to the uncertainty of the characteristics of the online respondents, in particular the lack of clinical confirmation of diagnosis [18]. Another study has demonstrated comparability in the findings derived from online blogs and face-to-face interviews, however [8].

Unlike the GCM and SMR, the CE interviews provided the opportunity for dialogue. As such, the interviewer was able to probe around the importance of concepts until the point at which the interviewer and interviewee felt that all symptoms had been explored fully. In this sense, the CE interview method is arguably unique in its capacity to glean the situated nature of symptoms in the context of subjects’ lives. That said, eight symptoms included in the combined conceptual model would have been missed if the CE interview data had been used in isolation.

Mixed methods approaches, such as GCM, are particularly beneficial because they combine qualitative and quantitative data. In the context of PRO research, this type of study is often performed with the aim of identifying concepts of interest. Thus, GCM may provide added value when there is a need to establish the relative importance of concepts in a way that gives instrument developers greater objectivity and confidence in measuring the most important concepts. However, if the aim of the study is to simply explore subjects’ perspectives to generate a better understanding of their broad disease experiences, then a qualitative study design (such as CE interviews), or a combination of the two, may be more appropriate.

### Which method had the most practical value?

The practical value of each method was considered in terms of time and budget as well as any logistical challenges. In pharmaceutical drug development, increased time is likely to equate to increased financial investment. The most time-efficient method to fully implement was the SMR as data collection could commence instantly upon finalization of the protocol, and subject recruitment was not required. In addition, the SMR focussed on English-speaking forums associated with a trust, charity or organization; thus, extracted posts were primarily from Western countries (i.e. North America, UK). Conducting SMR research in Non-Western countries would require special consideration regarding issues of linguistic and cross-cultural translation, and access to social media sites. Another important practical issue with SMR is how best to report the data in an ethical manner. In this paper, we refrained from reporting quotes as the authors could potentially be identified from these, and strongly encourage other researchers to follow suit. However, in a regulatory context, documentation of evidence for review is highly important; therefore, extra efforts to protect confidential information in submissions should be made, both in terms of internal reporting and submission documentation.

The CE interview method was the most time-intensive approach, although this could be mitigated somewhat by utilizing online or telephone interviews. In studies involving stakeholders from multiple institutions (where numerous and different review processes may be necessary) the time required could be substantially longer than in this study. Additionally, funds are often required to facilitate recruitment of subjects for primary research and these costs can quickly escalate, particularly in hard-to-reach patient groups (e.g. individuals with rare diseases). Importantly, of the three methods, CE interview is the only currently accepted by regulatory agencies.

For GCM, there was a logistical barrier in ensuring subjects had an appropriate computer device at home to complete the exercise. However, GCM can also be performed by hand using a paper-based format and hand sorting, albeit in a less efficient manner (e.g., requires interaction with a researcher and manual imputation of data).

### Which method appeared to be most engaging from a subject perspective?

Study subjects were recruited into a single study arm, so they were unable to indicate their preference of a particular method. However, feedback from participants indicated that they felt engaged in both the GCM exercise and the CE interviews. Regarding CE interviews, the face-to-face format meant that subjects had the opportunity to discuss their condition in-depth with the researcher. This was not only a point of satisfaction for the subjects, but had wider positive implications for the integrity of the data produced. As discussed previously, the interviewer had the opportunity to probe the subject about the severity and frequency of symptoms as well as the occurrence of “flare-ups.” Arguably, additional information about the variance in symptom experience can lead to a more comprehensive picture about the meaningfulness of symptoms to subjects’ lives. In the context of PRO development, understanding the relationship between symptoms and impacts is crucial.

One concern about using online data collection platforms is that it places too much distance between the researcher and subject. Yet, most subjects completing the GCM exercise did not report any issues. Conversely, subjects commented that the tasks were easy to complete and straightforward; although one subject reported that they would have liked some assistance with the “sorting” stage. A key benefit of GCM is that the conceptual model (or concept map) is participant-generated/authored as opposed to a researcher-led process. Consistent with this, our GCM subjects commented that they “*appreciated the chance to lead the research.*”

Although subjects’ feedback regarding the SMR in this study could not be obtained, in general, there appears to be a positive attitude among patients toward using social media data in outcomes research. In 2012, the Health Research Institute conducted a survey of 1060 US adult consumers and found that a third of respondents “*would be comfortable having their social media conversations monitored if it were to help improve their health, treatment coordination of care, or management of their chronic illnesses*.” [25] The success of social media sites such as PatientsLikeMe® also points to individuals’ willingness to share their health-related experiences if it improves their ability to self-manage and helps healthcare professionals better care for their patients.

### Study limitations

This prospectively designed study was conducted by a small researcher-led team with very few external factors to account for. In a more naturalistic research setting, there may be multiple stakeholders involved, often from a range of institutions. Therefore, the number of external variables that may impact the research process may be greater than we experienced in this study. In that sense, our experience of time and cost associated with the three research methods employed may be somewhat artificial and this should be accounted for when considering the real-world investment. One researcher analyzed the concept elicitation data; while the final thematic code list was verified by the project lead; a full independent analysis of the data could have further reduced any bias.

While subjects recruited for the GCM and CE interview provided basic demographic and clinical information during screening and were recruited via the membership of NASS, all diagnoses were self-reported and none were corroborated by the subject’s clinician. Morevoer, a major criticism of using secondary social media data in this type of research is related to the uncertainty of the characteristics of the online respondents, in particular the lack of clinical confirmation of diagnosis [18]. Another study has demonstrated comparability in the findings derived from online blogs and face-to-face interviews, however [20]. It would be important to replicate this study using subjects with a clinically confirmed diagnosis and compare their insights with those collected from social media sources to enable conclusions to be drawn around the “representativeness” and “validity” of secondary online data versus primary, prospective data. It would also be beneficial to validate the conceptual model directly with disease experts to confirm whether the symptoms represent those that are most critical based on their clinical experience of treating and managing patients with AS.

## Conclusions

This study aimed to explore the benefits and limitations of emerging methods for eliciting and collecting the subject’s perspective relative to traditional methods. We evaluated three approaches: 1) qualitative CE interviews, 2) a social media review, and 3) online data collection using GCM. Primary CE interviews achieved the greatest depth in conceptual understanding of patient experience; however, novel methods (GCM, SMR) provide complementary approaches for identifying measurement concepts. This study highlights that each approach has strengths and weaknesses and should be selected based on the research aims and context. Indeed, our findings demonstrate that the most complete conceptualization of the disease in question came when data from all three methods were combined. The combined conceptualization of the disease demonstrated that in addition to the cardinal symptoms of the condition which are well-reported in the literature, subjects also experienced other important symptoms that have not traditionally been considered part of the core disease.

An important next step will be to compare these different methods in the context of full PRO instrument development. At this stage, we have only considered the value and role of the different approaches when seeking to elicit early patient insight. An expansion of this study would be to compare the content of variations of a PRO measure that have been generated using data derived from different sources. Based our initial findings, it is likely that while variability would exist, the extent to which this would significantly misrepresent the patient’s experience appears to be minimal, thus supporting the use of novel pragmatic approaches in clinical outcomes research and in PRO measure development. This study is one of the first empirical, prospective evaluations of traditional versus creative methods for patient insights research; it should be viewed as an important step forward in the debate around the acceptance of such methods in future patient outcomes research and strategy.

## Additional file

Additional file 1: Table S1. List of statements generated by the GCM participants. (DOCX 13 kb)
