# Supplementary material for: A comparison of three methods to generate a conceptual understanding of a disease based on the patients’ perspective
Source: J Patient Rep Outcomes. 2017 Dec 19;1:9. doi: 10.1186/s41687-017-0013-6 (PMC5934934; doi:10.1186/s41687-017-0013-6)
Supplement: Supplementary file 1 — List of statements generated by the GCM participants. (DOCX 13 kb) [file 41687_2017_13_MOESM1_ESM.docx]

Table S1: List of statements generated by the GCM participants

*Note that some statements (eg, “anxiety” and “feeling depressed” are not present in the conceptual model after the research team’s interpretation that this would be categorised as an impact of AS rather than a symptom.*

| **#** | **Statement** |
| --- | --- |
| 1 | Fatigue |
| 2 | Stiff pelvis |
| 3 | Difficulty standing comfortably |
| 4 | Depression |
| 5 | Intermittent pain in back |
| 6 | General muscle aches |
| 7 | Intermittent pain in ankles |
| 8 | Intermittent pain in hands |
| 9 | Sacro-iliac joint pain |
| 10 | Stiff back |
| 11 | Difficulty sleeping |
| 12 | Aching hips |
| 13 | Feeling depressed |
| 14 | Stiff neck |
| 15 | Intermittent pain in neck |
| 16 | Intermittent pain in rib cage |
| 17 | Swollen knees |
| 18 | Muscle spasms in hamstrings |
| 19 | Dizziness |
| 20 | Aching shoulders |
| 21 | Pain and swelling when I smoke |
| 22 | Difficulty concentrating |
| 23 | Muscle tension |
| 24 | Stiff shoulders |
| 25 | Difficulty extending arms |
| 26 | Intermittent pain in achilles heel |
| 27 | Muscle spasms in spine |
| 28 | Muscle spasms in lower back |
| 29 | Severely restricted movement in neck |
| 30 | Clicking sounds in shoulder |
| 31 | Stiff torso |
| 32 | Generally feeling unwell/run down |
| 33 | Limited neck rotation |
| 34 | Limited spinal rotation |
| 35 | Sore knuckles |
| 36 | Neck pain |
| 37 | Problems with digestive system |
| 38 | Stiff jaw |
| 39 | Increased pain in cold/damp conditions |
| 40 | General stiffness |
| 41 | Cold or flu like symptoms |
| 42 | Uveitis |
| 43 | Intermittent pain in knees |
| 44 | Pressure in head |
| 45 | Pain in the morning |
| 46 | Shoulder pain when driving |
| 47 | Pain in back |
| 48 | Restricted forward movement in spine |
| 49 | Anxiety |
